# Targeted next generation sequencing for molecular diagnosis of Usher syndrome

**DOI:** 10.1186/s13023-014-0168-7

**Published:** 2014-11-18

**Authors:** María J Aparisi, Elena Aller, Carla Fuster-García, Gema García-García, Regina Rodrigo, Rafael P Vázquez-Manrique, Fiona Blanco-Kelly, Carmen Ayuso, Anne-Françoise Roux, Teresa Jaijo, José M Millán

**Affiliations:** Grupo de Investigación en Enfermedades Neurosensoriales. Instituto de Investigación Sanitaria IIS-La Fe, Semisótano Escuela de Enfermería, Hospital Universitario La Fe, Avda. Campanar, 21, 46009 Valencia, Spain; CIBER de Enfermedades Raras (CIBERER), Valencia, Spain; Servicio de Genética, IIS - Fundación Jiménez Díaz, University Hospital, UAM, Madrid, Spain; CHU Montpellier, Laboratoire de Génétique Moléculaire and Inserm, U827, Montpellier, F-34000 France; Unidad de Genética y Diagnóstico Prenatal, Hospital Universitario y Politécnico La Fe, Valencia, Spain

**Keywords:** Usher syndrome, Molecular diagnosis, Next generation sequencing, Point mutations, Large rearrangements

## Abstract

**Background:**

Usher syndrome is an autosomal recessive disease that associates sensorineural hearing loss, retinitis pigmentosa and, in some cases, vestibular dysfunction. It is clinically and genetically heterogeneous. To date, 10 genes have been associated with the disease, making its molecular diagnosis based on Sanger sequencing, expensive and time-consuming. Consequently, the aim of the present study was to develop a molecular diagnostics method for Usher syndrome, based on targeted next generation sequencing.

**Methods:**

A custom HaloPlex panel for Illumina platforms was designed to capture all exons of the 10 known causative Usher syndrome genes (*MYO7A*, *USH1C*, *CDH23*, *PCDH15*, *USH1G*, *CIB2*, *USH2A*, *GPR98*, *DFNB31* and *CLRN1*), the two Usher syndrome-related genes (*HARS* and *PDZD7*) and the two candidate genes *VEZT* and *MYO15A.* A cohort of 44 patients suffering from Usher syndrome was selected for this study. This cohort was divided into two groups: a test group of 11 patients with known mutations and another group of 33 patients with unknown mutations.

**Results:**

Forty USH patients were successfully sequenced, 8 USH patients from the test group and 32 patients from the group composed of USH patients without genetic diagnosis. We were able to detect biallelic mutations in one USH gene in 22 out of 32 USH patients (68.75%) and to identify 79.7% of the expected mutated alleles. Fifty-three different mutations were detected. These mutations included 21 missense, 8 nonsense, 9 frameshifts, 9 intronic mutations and 6 large rearrangements.

**Conclusions:**

Targeted next generation sequencing allowed us to detect both point mutations and large rearrangements in a single experiment, minimizing the economic cost of the study, increasing the detection ratio of the genetic cause of the disease and improving the genetic diagnosis of Usher syndrome patients.

## Background

Usher syndrome (USH) is an autosomal recessive disease characterized by the association of sensorineural hearing loss, retinitis pigmentosa (RP) and, in some cases, vestibular dysfunction. Its prevalence ranges from 3 to 6.2 per 100,000 [1–3] and it is the most common form of hereditary syndromes combining hearing loss and retinitis pigmentosa.

USH has a remarkable clinical and genetic heterogeneity. According to the severity and progression of the disease, three clinical types are distinguished. Type I (USH1) is defined by profound congenital hearing loss, onset of RP usually within the first decade of life and an absence of vestibular function. Usher syndrome type II (USH2) patients display congenital moderate-severe hearing loss, onset of RP around or after puberty and normal vestibular function. Usher syndrome type III (USH3) is characterized by postlingual progressive hearing loss, RP with a variable age of onset and variable vestibular response [4]). However, in some patients the disease does not fit into any of these three subtypes, and they are classified as ‘atypical Usher syndrome’.

To date, 10 genes and three additional loci have been associated with the disease. For USH1 six genes have been identified: *MYO7A* (USH1B), *USH1C* (USH1C), *CDH23* (USH1D), *PCDH15* (USH1F), *USH1G* (USH1G) and *CIB2* (USH1J); and two additional loci have been described: USH1E [5] and USH1H [6]. Three genes have been found to cause USH2: *USH2A* (USH2A), *GPR98* (USH2C) and *DFNB31* (USH2D). For USH3 only mutations in *CLRN1* have been described, and a second locus was proposed, USH3B. Furthermore, most of these genes are also responsible for non-syndromic hearing loss or isolated RP [4,7].

In addition to these 10 genes, three other genes have been associated with USH. *PDZD7* was proposed as a contributor of digenic inheritance with *GPR98*, and as a retinal disease modifier in *USH2A* patients [8]. Recently, a novel biallelic missense variant in *HARS* was identified in two patients with a phenotype compatible with USH3. This gene encoding an aminoacyl tRNA synthetase was proposed as a novel gene causative of USH3 [9]. More recently, *CEP250* has been associated with atypical Usher syndrome [10].

Proteins encoded by Usher genes belong to different classes: actin-based motor protein, scaffolding proteins, cell-adhesion molecules or transmembrane proteins, some of them with very large extracellular domains. These proteins through their protein-protein interacting domains are integrated in a network known as “Usher-interactome”. The main sites of colocalization of Usher proteins are the stereocilia or hair bundle of the inner ear hair cells, and the synaptic and periciliary areas of the photoreceptors [4,11].

In the stereocilia, two different USH protein networks are known to be involved in the development and maintenance of the inner ear hair cells. One of them is composed of the USH2 proteins, myosin VIIA (encoded by *MYO7A*), Vezatin and PDZD7 [12,13]. Vezatin is encoded by *VEZT* and it is required for the sound resilience of cochlear hair cells. In addition, it has been proposed that it is involved in the maturation steps of these cells or in the maintenance of junction integrity between hair cells and supporting cells in the inner ear [14]. The second stereocilia interactome is composed of the USH1 proteins [15]. In this network, several unconventional myosins, as myosin XVa, have also been found [16–18]. Mutations in *MYO15A*, the gene encoding Myosin XVa, are responsible for non-syndromic autosomal recessive hearing loss DFNB3 [19]. In addition, Myosin XVa interacts with whirlin in stereocilia, considered a key event in hair-bundle morphogenesis [20], and this direct interaction at the stereocilia tip is likely to control the elongation of stereocilia [21].

In the retina, two similar molecular networks composed of USH2 and USH1 proteins have also been described. The USH2 protein network, localized at the periciliary ridge complex region, contributes to the trafficking of cargos moving from the inner segment to the outer segment of the photoreceptor cells through the connecting cilium. The USH1 interactome has been reported to localize at the membrane-membrane connection site between the outer segment of the photoreceptor cells and the calyceal processes in primates. This USH1 protein complex is thought to form an adhesion belt and would contribute to the daily renewal of photoreceptors outer segments [22–24].

Traditionally, the molecular diagnosis of Usher syndrome has been mainly based on Sanger sequencing [25,26]. However, the large size of most USH genes (above 350 exons in total) makes this technique expensive and time-consuming. Array-based mutation screenings (arrayed primer extension (APEX) technology) have become a rapid and efficient technique to detect previously described mutations, and a specific APEX-microarray for USH was developed [26]. However, most of USH mutations are private, so the low detection rate of the APEX-microarray for Usher syndrome hampers the use of this technology [27,28]. Furthermore, Copy Number Variants (CNVs) have been identified as an important cause of the disease in Usher syndrome. Deletions and duplications are screened by Multiplex Ligation-dependent Probe Amplification (MLPA), for which commercial probemix is only available for *USH2A* and *PCDH15*, or by array-Comparative Genomic Hibridization (a-CGH) [29–31].

In recent years, next generation sequencing (NGS) techniques have been developed further, permitting the whole genome, whole exome and targeted gene sequencing to be more feasible, and hence making the identification of diseased genes and the underlying mutations easier, rapid and cost-effective. The improvement of NGS has been especially useful in the diagnosis of genetically heterogeneous diseases, such as hearing loss or retinal dystrophies [32–35].

For Usher syndrome, several NGS methods have been developed. Licastro et al. [36] used two different approaches: whole exome sequencing with the use of the SOLiD system and long-PCR sequencing on nine USH genes with two different platforms, Illumina (Genome Analyzer II) and the Roche 454 (GS FLX). Recently, Besnard et al. [37] developed a targeted NGS approach. They included 9 USH genes, 2 candidate USH genes (*VEZT* and *PDZD7*), seven hearing-loss genes and the choroideremia-causative gene *CHM* and the sequencing was carried out on Roche GS Junior sequencer. Yoshimura et al. [38] has also applied a massively parallel DNA sequencing methodology, but only for USH1 patients. Recently, Rong W et al. [39] used the NGS approach to identify three new alleles and one known mutation in *MYO7A* in three Chinese families.

We developed a molecular diagnosis of Usher syndrome based on a targeted NGS technique, HaloPlex (Agilent) gene target enrichment for Illumina platforms, including the ten USH known genes and four candidate genes that allowed us to identify not only point mutations but also CNVs, implementing a platform for the genetic diagnosis of this disease.

## Methods

### Patients

A cohort of 44 patients diagnosed with Usher syndrome was selected for this study. Their clinical classification into USH1, USH2, USH3 or atypical USH was performed on the basis of their clinical history and ophthalmologic, audiometric and vestibular tests. Patients included in this work were divided into two groups: a test group and a group composed by USH patients without genetic diagnosis.

Whenever possible, samples from additional family members were used to perform segregation analysis of the sequence variants identified in the index patient.

All patients and relatives included in the present study signed authorizing their written informed consent. The study was approved by the institutional board of the Ethics Committee of the University Hospital La Fe.

### Test group

The test group was composed of 11 patients: five USH1, four USH2, one USH3 and one atypical USH. These patients had been screened using a combination of techniques as Sanger sequencing, APEX microarray for Usher syndrome or MLPA. Variants of different nature in six USH genes had been previously detected and were used as positive controls in the present study. Table 1 shows the previously identified changes in the test group.

**Table 1:** **Test group: patients carrying sequence variants in USH genes previously detected**

**Patient**	**Clinical type**	**Gene**	**Type variant**	**Nucleotide**	**Protein**	**Classification**
RP-808	USH1	*CDH23*	Intronic	c.6059-9G > A	-----	Pathogenic. UV4
		*USH1G*	Missense	c.387A > G	p.Lys130Glu	UV1
		*USH1G*	Missense	c.423G > A	p.Glu142Lys	UV2
RP-1145	USH2	*USH2A*	Frameshift duplication	c.10272-10274dup	p.Cys3425Phefs*4	Pathogenic. UV4
		*USH2A*	Nonsense	c.7854G > A	p.Trp2618*	Pathogenic. UV4
		*DFNB31*	Isocoding	c.117G > A	p.Val39Val	UV1
RP-1286^§^	USH1	*PCDH15*	Frameshift insertion	c.1304_1305insC	p.Thr436Tyrfs*12	Pathogenic. UV4
RP-1374	USH1	*PCDH15*	Nonsense	c.7C > T	p.Arg3*	Pathogenic. UV4
RP-1426	USH1	*MYO7A*	Missense	c.6610G > C	p.Ala2204Pro	Pathogenic
RP-1522	USH2	*USH2A*	Frameshift deletion	c.2299delG	p.Glu767Serfs*21	Pathogenic. UV4
		*CDH23*	Missense	c.1096G > A	p.Ala366Thr	UV2
RP-1608^§^	USH3	*USH2A*	Missense	c.9799 T > C	p.Cys3267Arg	Pathogenic. UV4
RP-1614	USH1	*MYO7A*	Frameshift deletion	c.6025delG	p.Ala2009Profs*32	Pathogenic. UV4
RP-1637	USH2	*USH2A*	Frameshift duplication	c.5540dupA	p.Asn1848Glufs*20	Pathogenic. UV4
		*USH2A*	Large deletion	Del IVS4_IVS9	p.Gly262Valfs*2	Pathogenic. UV4
RP-1757^§^	Atypical	*MYO7A*	In-frame deletion	c.655_660del	p.Ile219_His220del	Pathogenic. UV4
RP-1760	USH2	*USH2A*	Missense	c.2296 T > C	p.Cys766Arg	UV3

Samples that failed in the amplification process are marked with §.

### Cohort of USH patients previously unscreened

A cohort of 33 USH patients without genetic diagnosis was enrolled in this study. It was composed of 13 USH1 patients, 17 USH2 and 3 USH3 cases.

### DNA Samples

Genomic DNA from patients and relatives was extracted from EDTA blood using an automated DNA extractor (Magna Pure, Roche). DNA samples were purified with the “*QIAqick PCR Purification Kit*” following the manufacturer’s instructions. The concentration of the genomic DNA was determined with the “*Qubit dsDNA BR Assay Kit*” in the Qubit 2.0 fluorometer.

### Targeted next generation sequencing design

A custom HaloPlex panel was designed using Agilent’s SureDesign tool (www.agilent.com/genomics/suredesign) to capture all exons and 25 bp of intronic flanking regions of 14 genes. The target genes included in our work were the 10 causative Usher syndrome genes (*MYO7A*, *USH1C*, *CDH23*, *PCDH15*, *USH1G*, *CIB2*, *USH2A*, *GPR98*, *DFNB31* and *CLRN1*), two related USH genes (*HARS* and *PDZD7*) and two candidate genes *VEZT* and *MYO15A*, due to their involvement in the Usher interactome [14,20,21]. The novel *CEP250* [10] had not been associated with USH at the beginning of this study, so it was not included in our work.

The entire custom design was 481 targets with a total size of 132.763 Kb. The exons from the different isoforms included in this study are summarized in Table 2. The intronic region where the *USH2A* mutation c.7595-2144A > G [37] is located, was included in the design. The final probe design size was 296.74 Kb with a theoretical coverage of 99.88% for our targeted regions. The difference in size between our entire custom design and the final probe design was due to an intrinsic process of the engineering of probes by Agilent’s Sure Design tool for the HaloPlex protocol.

**Table 2:** **Details about the exons studied in this targeted NGS analysis for the 14 genes analyzed**

**Chr**	**Gene**	**RefSec**	**Coding exons**	**Size (bp)**	**Coding Size (bp)**	**Amino acids**	**Alternative Ref Sec**	**Additional exons and the intronic** ***USH2A*** **region included in the study**	**Size (bp)**	**Number of exons analyzed**
11	*MYO7A*	NM_000260.3 variant 1	48	7465	6648	2215				48
10	*CDH23*	NM_022124.5 variant 1	69	11134	10065	3354	NM_052836.3 variant 2	1	150	70
10	*PCDH15*	NM_033056.3 variant C	32	7021	5868	1955	NM_001142773.1 variant H	1	6	38
							NM_001142769.1 variant I	2	871	
							NM_001142771.1 variant K	2	4469	
11	*USH1C*	NM_153676 variant 3	27	3246	2700	899	NM_005709.3 variant 1	1	75	28
17	*USH1G*	NM_173477.4 variant 1	3	3565	1386	461				3
15	*CIB2*	NM_006383.2 variant 1	6	1580	564	187				6
1	*USH2A*	NM_206933.2 variant 2	71	18883	15609	5202	NC_000001.11	c.7595-2144A > G	152	71 + 1 intronic sequence
5	*GPR98*	NM_032119.3 variant 1	90	19333	18921	6307				90
9	*DFNB31*	NM_015404.3 variant 1	12	4079	2724	907	NM_001083885.2 variant 2	1	110	13
3	*CLRN1*	NM_174878.2 variant 1	3	2359	699	232	NM_052995.2 variant 4	1	20	4
10	*PDZD7*	NM_001195263.1 variant 1	17	4164	3102	1033	NM_024895.4 variant 2	1	285	18
5	*HARS*	NM_002109.5 variant1	13	2322	1530	509				13
17	*MYO15A*	NM_016239.3	65	11876	10593	3530				65
12	*VEZT*	NM_017599 variant 1	12	4580	2340	779	NR_038242.1 variant 2	1	74	13
Total targets										481

### Sequence capture and next generation sequencing

Sequence capture was performed according to the “*HaloPlex Target Enrichment System*” (Protocol Version D.5, *Agilent Technologies Inc, CA, USA*) for Illumina Sequencing. Between 225 to 450 ng of gDNA were digested by 16 different restriction enzymes to create a library of gDNA restriction fragments. Four 12-reaction runs were performed including in each of them 11 gDNA samples and one Enrichment Control DNA sample. These enzymatic digestions were validated by electrophoretic analysis in a polyacrylamide gel. The gDNA restriction fragments were hybridized to the HaloPlex probe capture library. In this step, the Illumina sequencing motifs including index sequences were incorporated into the targeted fragments and the target DNA-HaloPlex probe hybrids were circularized. These hybrid molecules were captured using streptavidin beads. The DNA ligase was then added to close nicks in the circularized HaloPlex probe-target DNA hybrids and the captured DNA libraries were eluted with NaOH.

A PCR amplification of the captured target libraries was performed following the manufacturer’s instructions and, after its purification with the “*AMPure XP beads” (BECKMAN CULTER Inc)*, the validation and quantification of the enriched target DNA in each library was performed using the 2100 Bioanalyzer system with the *High Sensitivity DNA Kit* and the 2100 Expert Software (*Agilent Technologies Inc, CA, USA*). The last step of the protocol was to pool samples for multiplexed sequencing in the Illumina sequencing platform MiSeq System (*Illumina,Inc*).

### Variant analysis

Data was analyzed using the platform provided by DNAnexus (www.dnanexus.com). Two different versions were used: DNAnexus Classic and the DNAnexus version recently implemented. Annotated variants were selected according to the following criteria: the quality value should be ≥250, a percentage of heterozygosity ≥30% of the reads, their annotation in the dbSNP (http://www.ncbi.nlm.nih.gov/SNP) and their description in the Usher syndrome mutation database (https://grenada.lumc.nl/LOVD2/Usher_montpellier). Variants should be observed in both direct and reverse strands.

Variants selected and suspected to be pathogenic were confirmed by Sanger sequencing. The DNA fragments containing the variants were amplified by PCR with specific primers and were sequenced on both strands using the Big Dye 3.1 Terminator Sequencing Kit. The purified sequence products were analyzed on a 3500xl ABI instrument (*Applied Biosystems by Life Technologies, Thermo Fisher Scientific, Inc*).

The pathogenicity of novel missense variants was analyzed with the SIFT (http://sift.bii.a-star.edu.sg/) and PolyPhen-2 (http://genetics.bwh.harvard.edu/pph2/) algorithms. Those putative variants that affect the splicing process were studied with the NetGene2 (http://www.cbs.dtu.dk/services/NetGene2), NNSPLICE v0.9 (http://www.fruitfly.org/seq_tools/splice.html), Human Splicing Finder (HSF; http://www.umd.be/HSF) and RESCUE-ESE (http://genes.mit.edu/burgelab/rescue-ese/) programs.

Novel variants were classified based on the classification system for Unknown Variants (UV) (https://grenada.lumc.nl/LOVD2/Usher_montpellier) as pathogenic (UV4), possibly pathogenic (UV3), possibly non-pathogenic (possibly neutral, UV2) and non-pathogenic (neutral, UV1) according to bioinformatics predictions and segregation analysis. This classification is in line with the guidelines published by the clinical and molecular genetics society (http://cmgdweb.shared.hosting.zen.co.uk/BPGs/Best_Practice_Guidelines.htm).

Nomenclature of variants was performed according to the reference sequences: *MYO7A* (NM_000260.3), *USH1C* (NM_153676), *CDH23* (NM_022124.5), *PCDH15* (NM_033056.3), *USH1G* (NM_173477), *CIB2* (NM_006383.2), *USH2A* (NM_206933), *GPR98* (NM_032119.3), *DFNB31* (NM_015404), *CLRN1* (NM_174878), *HARS* (NM_002109), *PDZD7* (NM_001195263.1), *VEZT* (NM_017599) and *MYO15A* (NM_016239).

Segregation analysis was performed in cases where DNA samples of relatives were available.

### Copy number variation analysis and validation

The coverage of every target region of the sample of interest was normalized and compared with average normalized data of all other samples of the same run to obtain the ratio relative coverage. Deletions and duplications were suspected of being present if this ratio fell below 0.7 or rose above 1.3 respectively.

Validation of rearrangements of *USH2A* and *PCDH15* was performed by MLPA analysis. For *USH2A* the SALSA MLPA probemixes P361 and P362 were used. To confirm the *PCDH15* copy number variations the SALSA MLPA probemix P292 was employed. The MLPA reactions were performed according to the manufacturer’s recommendations (http://www.mlpa.com).

In the cases of putative rearrangements identified in *CDH23* and *GPR98*, their validation was carried out by an oligonucleotide array-CGH. The array-CGH chip included 77,366 probes covering the genes *MYO7A*, *CDH23*, *PCDH15*, *USH1C*, *USH1G*, *USH2A*, *GPR98*, *DFNB31*, *PDZD7* and *CLRN1* and 10.000 nucleotides of 5′ and 3′ untranslated regions [30].

## Results

### Next generation sequencing results of the USH panel

**Figure 1:**
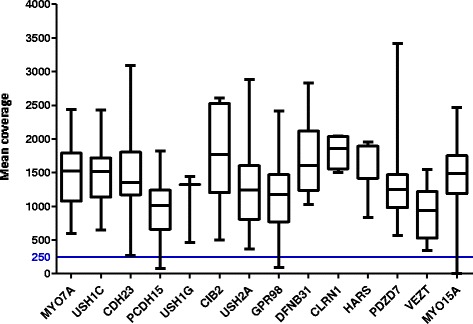
**Mean coverage obtained for the different genes.** The blue line shows the lower limit of coverage appropriate to perform CNV analyses (250x).

Capture of NGS using our customized USH panel was performed in a cohort of USH patients. High quality results were obtained. On average, a mean coverage of 1334x was obtained per sample and target region. Different average coverages were obtained for the fourteen analyzed genes, ranging from 935x (*VEZT*) to 1817x (*CLRN1*) (Figure 1).

**Figure 2:**
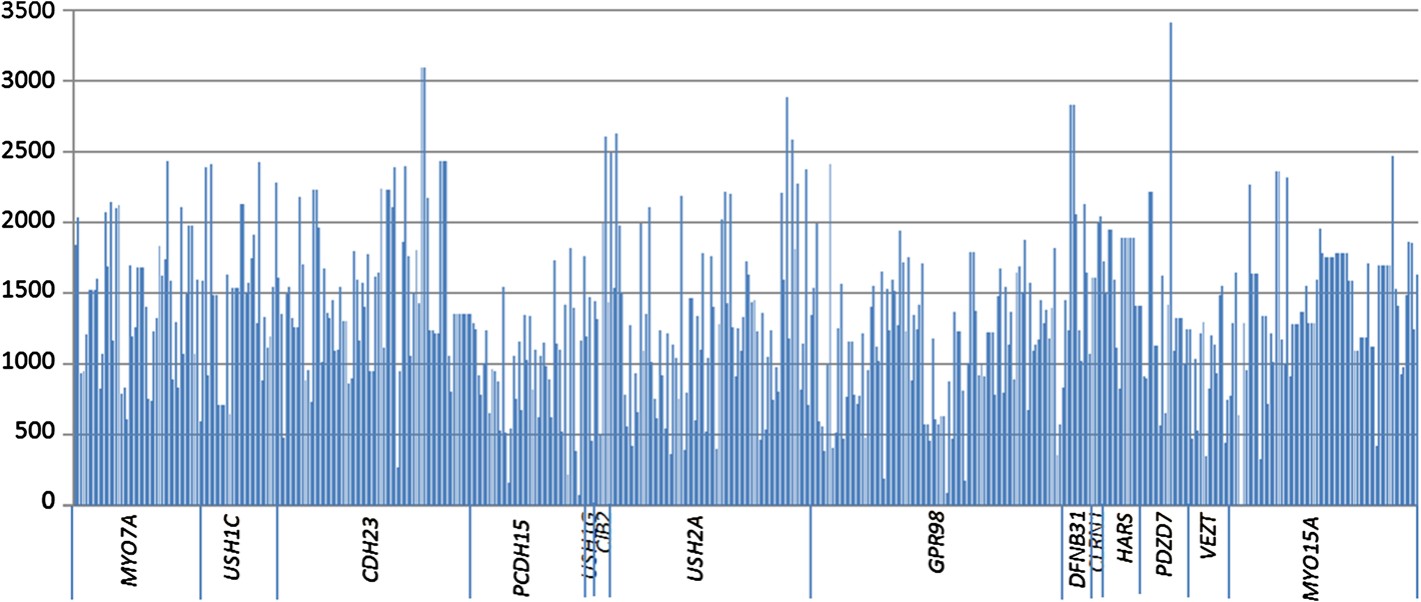
**Mean coverage of all targeted regions included in our custom design.**

Only one of the selected targeted regions showed coverage lower than 40x, the defined limit for proper validation and diagnostic procedures. This target was a coding region of 43 bp in the large exon 2 of *MYO15A*. A schematic representation of the coverage of all target regions is showed in Figure 2.

### Test group: validation of the diagnostic strategy

Eleven USH patients were included in the test group to verify the reliability of our NGS custom panel. In these cases, 17 mutations and polymorphism had been previously detected in different USH genes by Sanger or by MLPA (Table 1).

During the experiment three control patients and one studied patient failed in the capture step of the target DNA of the HaloPlex protocol. Due to a manual/technical problem with the PCR strips and magnetic rack Dynabeads, the circularized target DNA-HaloPlex probe hybrids, containing biotin, were not captured on streptavidin beads and these samples were lost.

The remaining eight patients were carriers of 14 different variants including missense, nonsense, point deletions or insertions and large deletions (Table 1). Our NGS approach allowed us to detect all previously detected changes in the testing cohort, both point mutations and CNVs.

The sequencing of the USH genes allowed the detection of a second pathologic allele in five patients in whom only one mutation was previously detected. In addition, in patient RP-1426, previously a carrier of a pathogenic variant in *MYO7A,* we were able to identify a novel splice site mutation in *CDH23*. These results are summarized in Table 3.

**Table 3:** **Causative mutations and putative pathogenic variants identified in this study**

**Patient**	**Clinical type**	**Gene**	**Exon**	**Nucleotide variant**	**Protein variant**	**Reference**	**Segregation analysis**
**Patients with two pathogenic mutations in the same gene**							
RP-807	USH2	*MYO7A*	40	**c.5516 T > C**	**p.Leu1839Pro**	Novel. UV3	Yes
		*MYO7A*	27	**c.3503G > A**	**p.Arg1168Gln**	Novel. UV3	
RP-808^¶^	USH1	*CDH23*	47	c.6059-9G > A	---	von Brederlow et al., (2002) [40]	No
		*CDH23*	10	**c.871G > A**	**p.Gly291Arg**	Novel. UV3	
RP-890	USH3	*USH2A*	26	c.5278delG	p.Asp1760Metfs*10	Garcia-Garcia et al., (2011) [41]	No
		*USH2A*	26	c.5278delG	p.Asp1760Metfs*10	Garcia-Garcia et al., (2011) [41]	
RP-1182	USH1	*PCDH15*	22_23	**Duplication exons 22_23**	---	Novel. UV4	No
		*PCDH15*	22_23	**Duplication exons 22_23**	---	Novel. UV4	
RP-1183	USH1	*CDH23*	26	c.3016G > A	p.Glu1006Lys	Schultz et al., (2011) [42]	No
		*CDH23*	26	c.3016G > A	p.Glu1006Lys	Schultz et al., (2011) [42]	
RP-1234	USH1	*MYO7A*	43	**c.5884delTTCT**	p.Phe1962Leufs*7	Novel. UV4	Yes
		*MYO7A*	43	**c.5884delTTCT**	p.Phe1962Leufs*7	Novel. UV4	
RP-1237	USH1	*CDH23*	46	c.6049G > A	p.Gly2017Ser	Roux et al., (2006) [43]	No
		*CDH23*	46	c.6049G > A	p.Gly2017Ser	Roux et al., (2006) [43]	
RP-1374^¶^	USH1	*PCDH15*	2	c.7C > T	p.Arg3*	Ahmed et al., (2001) [44]	Yes
		*PCDH15*	27	c.3717 + 2dupT	---	Jaijo et al., (2012) [45]	
RP-1422	USH1	*MYO7A*	43	c.5944G > A	p.Gly1982Arg	Riazuddin et al., (2008) [46]	Yes
		*MYO7A*	43	c.5944G > A	p.Gly1982Arg	Riazuddin et al., (2008) [46]	
RP-1522^¶^	USH2	*USH2A*	13	c.2299delG	p.Glu767Serfs*21	Liu et al., (1999) [47]	No
		*USH2A*	20	**Deletion exon 20**	**---**	Novel. UV4	
RP-1551	USH1	*PCDH15*	27	**c.3511delA**	p.Asp1172Ilefs*13	Novel. UV4	Yes
		*PCDH15*	27	**c.3511delA**	p.Asp1172Ilefs*13	Novel. UV4	
RP-1614^¶^	USH1	*MYO7A*	44	c.6025delG	p.Ala2009Profs*32	Bharadwaj et al., (2000) [48]	No
		*MYO7A*	40	**c.5537C > A**	**p.Pro1846His**	Novel. UV3	
RP-1760^¶^	USH2	*USH2A*	55	**c.10888delA**	p.Gly3631Valfs*43	Novel. UV4	No
		*USH2A*	13	c.2296 T > C	p.Cys766Arg	Glöcke et al., (2013) [35]	
RP-1781	USH2	*CDH23*	29	**Duplication exon 29**	---	Novel. UV4	No
		*CDH23*	68	**c.9569C > T**	**p.Ala3190Val**	Novel. UV3	
RP-1791	USH1	*MYO7A*	20	c.2283-1G > T	---	Roux et al., (2006) [43]	Yes
		*MYO7A*	28	c.3594C > A	p.Cys1198*	Roux et al., (2011) [43]	
RP-1802	USH2	*USH2A*	63	c.13811 + 2 T > G	---	Besnard et al., (2014) [37]	No
		*USH2A*	50	c.9799 T > C	p.Cys3267Arg	Aller et al., (2006) [49]	
RP-1835	USH2	*USH2A*	57	c.11065C > T	p.Arg3689*	Le Quesne Stabej et al., 2012 [50]	Yes
		*USH2A*	22	**c.4758 + 3A > G**	**---**	Novel. UV3	
RP-1864	USH2	*MYO7A*	6	c.494C > T	p.Thr165Met	Ouyang et al., (2005) [51]	No
		*MYO7A*	6	c.494C > T	p.Thr165Met	Ouyang et al., (2005) [51]	
RP-1895	USH2	*GPR98*	79_83	Duplication exons 79_83	---	Besnard et al., (2012) [52]	No
		*GPR98*	79_83	Duplication exons 79_83	---	Besnard et al., (2012) [52]	
RP-1904	USH2	*GPR98*	11	**c.2145_2149delGTTTT**	p.Leu715Phefs*6	Novel. UV4	Yes
		*GPR98*	14	**c.2612delG**	p.Gly871Glufs*8	Novel. UV4	
RP-1910	USH1	*CDH23*	60	c.8722 + 1delG	---	Oshima et al., (2008) [53]	Yes
		*CDH23*	60	c.8722 + 1delG	---	Oshima et al., (2008) [53]	
RP-1924	USH1	*MYO7A*	39	c.5392C > T	p.Gln1798*	Janecke et al., (1999) [54]	Yes
		*MYO7A*	27	**c.3503G > A**	**p.Arg1168Gln**	Novel. UV3	
RP-1927	USH2	*USH2A*	21	c.4474G > T	p.Glu1492*	Bernal et al., (2005) [55]	Yes
		*USH2A*	2	**c.269A > G**	**p.Tyr90Cys**	Novel. UV3	
RP-1948	USH1	*MYO7A*	7	**C.707 T > A**	**p.Leu236Gln**	Novel. UV3	No
		*MYO7A*	42	c.5749G > T	p.Glu1917*	Jacobson et al., (2009) [56]	
RP-1960	USH2	*USH2A*	25	**c.5167G > C**	**p.Gly1723Arg**	Novel. UV3	No
		*USH2A*	7	c.1214delA	p.Asn405Ilefs*3	Bernal et al., (2005) [55]	
**Patients with three pathogenic mutations in two different genes**							
RP-1847	USH2	*USH2A*	62	c.12067-2A > C	---	Kaiserman et al., (2007) [57]	Yes
		*USH2A*	14	Deletion exon 14	---	Glöckle et al., (2013) [35]	
		*USH1G*	2	**c.805C > T**	p.Arg269*	Novel. UV4	
RP-1923	USH2	*USH2A*	62	c.12093delC	p.Tyr4031*	Garcia-Garcia et al., (2011) [41]	No
		*USH2A*	44	Deletion exon 44	---	Glöckle et al., (2013) [35]	
		*DFNB31*	9	**c.2234G > A**	**p.Arg745His**	Novel. UV3	
**Patients with only one pathogenic mutation**							
RP-1455	USH1	*USH2A*	28	**c.5666A > G**	**p.Asp1889Gly**	Novel. UV3	No
RP-1496	USH3	*GPR98*	19	**c.3443G > A**	**p.Gly1148Asp**	Novel. UV3	No
RP-1741	USH2	*USH2A*	PE40	c.7592-2144A > G	---	Vaché et al., (2012) [58]	No
RP-1929	USH2	*GPR98*	58	**c.11974G > A**	**p.Asp3992Asn**	Novel. UV3	No
RP-1953	USH2	*USH1C*	18	c.1859G > T	p.Arg620Leu	Ouyang et al., (2002) [59]	No
**Patients with pathologic mutations in different genes**							
RP-1426^¶^	USH1	*MYO7A*	49	c.6610G > C	p.Ala2204Pro	Jaijo et al., (2007) [60]	Yes
		*CDH23*	39	**c.5068-2A > T**	**---**	Novel. UV4	
RP-1950	USH2	*USH2A*	70	c.2299delG	p.Glu767Serfs*21	Liu et al., (1999) [47]	No
		*GPR98*	70	**c.14278C > T**	**p.Pro4760Ser**	Novel. UV3	

Patients previously included in the test group are marked with ¶.

Novel variants are marked in bold.

*PE*: Pseudoexon 40.

### Cohort of USH patients previously unscreened

In our cohort of 33 USH patients studied, mutations could be identified in the majority of analyzed cases. One of the USH cases failed in the capture and amplification of the library due to the same manual/technical error explained above. If we take into account the remaining 32 USH cases analyzed, we identified in 22 patients the two expected mutated alleles, in 5 cases only one pathogenic variant and in one case, RP-1950, two pathogenic variants were detected in two different USH2 genes. In four patients no mutation was found. In addition two patients (RP-1847 and RP-1923) were carriers of three mutations in USH genes (Table 3).

In this study, we were able to identify 53 different mutations, of which 24 were novel. These variants included 21 missense, 8 nonsense, 9 frameshifts, 9 intronic mutations and 6 large rearrangements.

The DNA nexus program showed a high number of novel variants in each patient. These changes were selected according to their nature, or the presence of another pathogenic allele in the same gene in the same patient. The variants were confirmed by Sanger sequencing and missense, isocoding or intronic variants were analyzed by different *in silico* algorithms to predict their pathogenic effect. According to the bioinformatics predictions, 15 variants out of 25 studied variants were considered as pathogenic (UV4) or possibly pathogenic (UV3) (Table 4).

**Table 4:** **Summary of putative pathogenic mutations and their bioinformatics predictions**

**Patient**	**Gene**	**Exon**	**Nucleotide Change**	**Amino Acid Change**	**Classification**	**SIFT (score)**	**PolyPhen-2 (score)**	**NetGene2**	**Human Splicing Finder**	**NNSPLICE**	**RESCUE-ESE**
RP-1948	*MYO7A*	7	c.707T>A	p.Leu236Gln	UV3	deleterious (0)	probably_damaging (1)	Neutral	Neutral	Neutral	A new ESE site is created
RP-807 RP-1924	*MYO7A*	27	c.3503G>A	p.Arg1168Gln	UV3	deleterious (0)	probably_damaging (1)	Score for the main donor site decreases from 95 to 75	Score for the main donor site decreases from 90 to 80	The main donor site is not recognized	Neutral
RP-807	*MYO7A*	40	c.5516T>C	p.Leu1839Pro	UV3	deleterious (0)	probably_damaging (1)	Score for the main acceptor site decreases from 80 to 77	Neutral	Neutral	Neutral
RP-1614	*MYO7A*	40	c.5537C>A	p.Prp1846His	UV3	deleterious (0.01)	possibly_damaging (0.85)	Score for the main acceptor site decreases from 80 to 77 and one acceptor site is not recognized	Neutral	Neutral	Neutral
RP-808	*CDH23*	10	c.871G>A	p.Gly291Arg	UV3	deleterious (0)	probably_damaging (1)	Neutral	Neutral	Neutral	A new ESE site is created
RP-1426	*CDH23*	39	c.5068-2A>T	c.5068-2A>T	UV4	---	---	The main acceptor site is not recognized	Score for the main acceptor site decreases from 95 to 66	The main acceptor site is not recognized	---
RP-1781	*CDH23*	68	c.9569C>T	p.Ala3190Val	UV3	deleterious (0)	probably_damaging (1)	Neutral	Neutral	Neutral	Neutral
RP-1927	*USH2A*	2	c.269A>G	p.Tyr90Cys	UV3	tolerated (0.17)	possibly_damaging (0.796)	Score for acceptor site decreases from 82 to 80	Neutral	Score for acceptor site decreases from 75 to 69	A ESE site is not recognized
RP-1835	*USH2A*	22	c.4758+3A>G	c.4758+3A>G	UV3	---	---	The main donor site is not recognized	Neutral	The main donor site decreases from 98 to 73	---
RP-1960	*USH2A*	25	c.5167G>C	p.Gly1723Arg	UV3	deleterious (0)	probably_damaging (0.994)	The main donor site is not recognized	Score for the main donor site decreases from 86 to 75	The main donor site is not recognized	Neutral
RP-1455	*USH2A*	28	c.5666A>G	p.Asp1889Gly	UV3	deleterious (0)	probably_damaging (0.982)	The main donor and acceptor sites decrease from 82 to 80 and from 53 to 48 respectively and a new acceptor site is created	Neutral	Neutral	Two ESEs are not recognized
RP-1496	*GPR98*	19	c.3443G>A	p.Gly1148Asp	UV3	deleterious (0)	probably_damaging (0.999)	Neutral	Neutral	Neutral	Neutral
RP-1929	*GPR98*	58	c.11974G>A	p.Asp3992Asn	UV3	deleterious (0.01)	probably_damaging (0.999)	Neutral	Neutral	Neutral	A ESE is not recognized
RP-1950	*GPR98*	70	c.14278C>T	p.Pro4760Ser	UV3	deleterious (0)	probably_damaging (0.998)	Neutral	Neutral	Neutral	Neutral
RP-1923	*DFNB31*	9	c.2234G>A	p.Arg745His	UV3	deleterious(0.01)	probably_damaging (0.984)	Neutral	Neutral	The main donor site increases from 66 to 86	Neutral

*SIFT*: SIFT Score ranges from 0 to 1. The amino acid substitution is predicted to be damaging if the score is < 0.05, and tolerated if the score is > 0.05.

PolyPhen stablish three classifications: “Probably damaging” (it is believed most likely to affect protein function or structure), “Possibly damaging” (it is believed to affect protein function or structure), “Benign” (most likely lacking any phenotypic effect).

*ESE*: Exonic Splicing Enhancer.

Segregation analysis of detected alleles with the respective family members was performed in 13 cases, and cosegregation of the mutations with the disease was verified in all these cases (Table 3).

### CNVs Detection

Next Generation Sequencing with the Illumina MiSeq system allowed us to perform qualitative and quantitative analysis. We could detect not only point mutations but also CNVs.

In our cohort of patients we performed CNV analysis in all patients where two point mutations had not been detected. This analysis allowed us to identify six large rearrangements: three deletions in *USH2A* comprising the exons 14, 20 and 44, and three duplications comprising the exon 29 in *CDH23*, exons 22_23 in *PCDH15* and exons 79_83 in *GPR98*. The *USH2A* and the *CDH23* rearrangements were detected in a heterozygous state, whereas the *PCDH15* and *GPR98* duplications were identified in a homozygous state.

Large rearrangements in *USH2A* and *PCDH15* were subsequently confirmed by MLPA, whereas patients that carried duplications affecting *CDH23* and *GPR98* were analyzed by a-CGH (data not shown). In 5 patients, the rearrangements detected by comparison of normalized coverage data were confirmed. The a-CGH technique to confirm the presence of the heterozygous duplication of *CDH23* (exon 29) in patient RP-1781 was not succesful due to technical problems. Unfortunately, it was impossible to get a new DNA sample for this patient to repeat the experiment.

Three of the six rearrangements found in this study had been previously described. The *USH2A* deletions of exon 14 and exon 44 were described by Glockle et al. [35] and the *GPR98* duplication was previously described by Besnard et al. [52]. The deletion of exon 14 in *USH2A* has also been detected by our group, in one Spanish USH patient in a homozygous state (unpublished results).

In our cohort of patients previously unscreened we could detect 51 altered alleles, six of which corresponded to large rearrangements, i.e. 11.76% of the detected pathogenic alleles.

**Figure 3:**
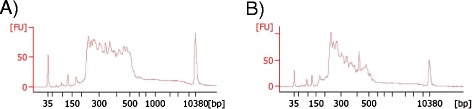
**Images obtained in the validation and quantification of the enriched target DNA with the 2100 Bioanalyzer system. A)** Typical image obtained in most patients. **B)** Atypical image obtained in the patient RP-531, in whom CNV analysis could not be performed.

In one patient no point mutation was identified, and the quantitative analysis could not be performed (RP-531). The ratio between this sample and the normalized data was altered in a high number of target regions from most genes. In this case, the image obtained with the bioanalyzer in the validation and quantification of the enriched target DNA process was atypical (Figure 3).

In some cases, we could observe that the CNV analysis displayed doubtful results in the targeted regions. The analysis of those target regions revealed coverages lower than 250x.

## Discussion

### Resolved cases

Usher syndrome displays both genetic and allelic heterogeneity. The number of genes related to the disease and the large size of most of them contribute to the genetic heterogeneity. Furthermore, mutations causing disease include point mutations and large rearrangements, most of them, being private. Recently, the high-throughput sequence analysis and next generation sequencing, has been proved to be very helpful to carry out genetic studies in heterogeneous diseases.

In this study we have developed a targeted method based on NGS for the molecular diagnosis of Usher syndrome. We evaluated our panel in a group of patients with known USH variants and applied it to a cohort of USH patients previously unscreened.

In our series of study, we were able to detect biallelic mutations in one USH gene in 22 out of 32 USH patients (68.75%) and to identify 51 out of the 64 expected mutated alleles (a detection ratio of 79.7%).

### Uncovered regions

We obtained a highly heterogeneous coverage using a custom *HaloPlex Target Enrichment System* (see Figure 1). However, only one region included in the final design showed a mean coverage lower than 40x in our samples. It corresponded to a 43 bp sequence in the large exon 2 of *MYO15A*. That fragment shows a high percentage of CGs, 77%. It has been reported that most sequencing platforms, including Illumina sequencers, show a GC content-dependent bias coverage, and protocols should be modified to minimize these events [61].

### Unresolved cases

Mutations were not detected in four patients of our cohort. In one USH1 case detailed clinical data could not be obtained, whereas in the remaining cases clinical information was revised and the USH diagnosis was confirmed. Five additional patients were carriers of only one pathogenic variant (Table 3), the second mutation remaining unidentified.

In these cases, in which the second mutated allele escapes detection, the region that contains the mutation may have been excluded from our study. It is possible that the gene responsible for the disease had not yet been identified, and therefore it could not be included in our panel. Furthermore, deep intronic regions, promoter regions or 5′ and 3′ untranslated regions were not systematically included in the panel design. The only intronic region targeted in our panel was designed to detect the *USH2A* c.7595-2144A > G mutation identified by Vaché et al. [58] in five French patients and four Spanish patients. Moreover, it was subsequently detected in five additional families [31]. Subsequently, Steele-Stallard et al. [31] detected this intronic change in three UK and European USH families.

In two patients, two mutated alleles, each one in a different gene, were detected (Table 3). Patient RP-1426 was carrier of a missense *MYO7A* variant (p.Ala2204Pro). Using the NGS panel we could not detect a second mutation in *MYO7A,* but a splice acceptor site mutation (c.5068-2A > T) was found in *CDH23*. In the USH2 patient RP-1950, the *USH2A* mutation c.2299delG and a novel missense variant (p.Pro4760Ser) in *GPR98* were detected. Bioinformatics algorithms predicted a pathogenic effect for these two novel variants (Table 4). In the former case, the two mutations were present in the same, clinically unaffected parent (data not shown), which does not support a digenic inheritance of USH. Unfortunately, segregation analysis could not be performed in the case of patient RP-1950. Perhaps a second mutation remains undetected in one of these genes in the patients RP-1426 and RP-1950. In two other patients (RP-1847 and RP-1923) with biallelic mutations in *USH*2A, we were able to detect additional mutations in USH1G and DFNB31, respectively. These cases show the high decisive power of massive parallel re-sequencing, detecting additional mutations in patients carrying the two mutations responsible for the disease in the same gene.

The programs used to analyze the data generated from NGS followed different algorithms. In fact, both the alignment and the base calling are two crucial steps in these analyses. It is possible that some mutations could escape detection due to the algorithms used. Their modification will improve the quality of generated data, avoiding false negatives and allowing detection of missed mutations in further analysis.

Four patients carried two mutations in genes typically responsible for another clinical subtype. Two USH2 patients carried missense mutations in *MYO7A (*RP-807 and RP-1864), one USH2 case in *CDH23* (RP-1781) and a USH3 patient carried a homozygous frameshift deletion in *USH2A* (RP-890) (Table 2). The situation of USH patients diagnosed with a given clinical subtype, who carry mutations in a gene involved in a different subtype, is not unprecedented, and was previously reported [25]. These findings further support the use of NGS techniques that allow for the study of all known causative genes for Usher syndrome in patients independent of their clinical subtype. It facilitates the detection of mutations in genes typically associated with other clinical types.

### Copy number variation analysis

Studies performed in different populations have demonstrated that large rearrangements are an important cause of the disease in Usher syndrome. Le Guedard et al. [62] and Aller et al. [29] demonstrated that *PCDH15* rearrangements are a significant cause of USH alterations. Large deletions involving *USH2A* have been reported [22,35], and large rearrangements in other USH genes as *MYO7A*, *CDH23* or *GPR98* have been detected [30,62]. These results indicate that the genetic analysis in USH patients should include the screening of rearrangements.

We have obtained with the MiSeq system a mean coverage in our USH panel of 1334x per sample and target region, allowing for CNV analysis of the selected genes. We could then detect in our series of patients six different rearrangements in *CDH23*, *PCDH15*, *USH2A* and *GPR98*, which represents 11.8% of the mutated detected alleles. We observed that when the coverage of regions fell below 250x, the results obtained in the CNV analysis were questionable and we did not take into account those regions. In order to perform quantitative analysis, we recommend, therefore, coverage of targeted regions above 250x.

In one patient (RP-531) the validation of the enrichment process gave an atypical image in the bioanalyzer (Figure 3). In this case the CNV analysis could not be performed. However, this does not affect the detection of point mutations. In fact, in another patient with a similar image in the validation (RP-808), two point mutations were detected.

### Comparison with other NGS studies of USH

Traditionally, Sanger sequencing was used to identify the mutations responsible of the disease [25,50]. Nowadays different methods based on NGS have been used for Usher syndrome. Licastro et al. [36] employed two different approaches: the whole exome sequencing and long-PCR sequencing on nine USH genes. Whole exome sequencing has several problems: a low coverage of the interesting regions and the need of a correct interpretation of the high number of sequence variations identified. Regarding the long-PCR method, it displayed a better mean coverage, higher than 25x in 94% of regions. Taking together both approaches, the detection of pathogenic alleles was 62.5%.

Recently, Besnard et al. [37] developed a targeted NGS panel where 9 USH genes, 2 candidate USH genes (*VEZT* and *PDZD7*), seven hearing-loss genes and *CHM* were included. The overall depth of coverage obtained was estimated as 77x across the whole design ranging from 55.8x to 106.9x, with a detection ratio of 84.6% in the studied USH cohort.

In our series, the detection rate of mutated alleles was 79.7%. Our results are close to the Besnard et al. [37] study and higher than to the Licastro et al. [36] approaches. However, it is difficult to obtain conclusions due to the low number of patients included in those studies (thirteen and twelve USH patients respectively), whereas in our series 32 USH patients previously unscreened were sequenced.

Furthermore, in both previous studies, only point mutations were detected. The mean coverage obtained did not allow for the analysis of CNVs, that has been observed as an important USH cause. As has been pointed by Eisenberger et al.[63], high and extensive coverage allows for systematic analysis for CNVs and reduces the risk of mutations escaping detection because of their localization in regions with low coverage.

## Conclusions

Our developed targeted NGS method based on HaloPlex gene target enrichment technology for the genetic diagnosis of Usher syndrome provides nearly complete coverage of all coding regions of the ten USH genes and four related and candidate USH genes. Furthermore, the high coverage obtained in our study allowed us to detect large rearrangements. It is important to detect both point mutations and large deletions or duplications with a single technique to minimize the economic cost of these studies, increasing the detection ratio of the genetic cause of the disease and improving the genetic diagnosis of Usher syndrome patients.

## Availability of supporting data

LOVD http://www.lovd.nl/2.0/index_list.php

## References

[1] Espinos C, Millan JM, Beneyto M, Najera C (1998). Epidemiology of Usher syndrome in Valencia and Spain. Community Genet.

[2] Keats BJ, Corey DP (1999). The Usher syndromes. Am J Med Genet.

[3] Cohen M, Bitner-Glindzicz M, Luxon L (2007). The changing face of Usher syndrome: clinical implications. Int J Audiol.

[4] Millán JM, Aller E, Jaijo T, Blanco-Kelly F, Gimenez-Pardo A, Ayuso C (2011). An update on the genetics of Usher syndrome. J Ophthalmol.

[5] Chaïb H, Kaplan J, Gerber S, Vincent C, Ayadi H, Slim R, Munnich A, Weissenbach J, Petit C (1997). A newly identified locus for Usher syndrome type I, USH1E, maps to chromosome 21q21. Hum Mol Genet.

[6] Ahmed ZM, Riazuddin S, Khan SN, Friedman PL, Riazuddin S, Friedman TB (2009). USH1H, a novel locus for type I Usher syndrome, maps to chromosome 15q22-23. Clin Genet.

[7] Riazuddin S, Belyantseva IA, Giese AP, Lee K, Indzhykulian AA, Nandamuri SP, Yousaf R, Sinha GP, Lee S, Terrell D, Hegde RS, Ali RA, Anwar S, Andrade-Elizondo PB, Sirmaci A, Parise LV, Basit S, Wali A, Ayub M, Ansar M, Ahmad W, Khan SN, Akram J, Tekin M, Riazuddin S, Cook T, Buschbeck EK, Frolenkov GI, Leal SM, Friedman TB (2012). Alterations of the CIB2 calcium- and integrin-binding protein cause Usher syndrome type 1 J and nonsyndromic deafness DFNB48. Nat Genet.

[8] Ebermann I, Phillips JB, Liebau MC, Koenekoop RK, Schermer B, Lopez I, Schäfer E, Roux AF, Dafinger C, Bernd A, Zrenner E, Claustres M, Blanco B, Nürnberg G, Nürnberg P, Ruland R, Westerfield M, Benzing T, Bolz HJ (2010). PDZD7 is a modifier of retinal disease and a contributor to digenic Usher syndrome. J Clin Invest.

[9] Puffenberger EG, Jinks RN, Sougnez C, Cibulskis K, Willert RA, Achilly NP, Cassidy RP, Fiorentini CJ, Heiken KF, Lawrence JJ, Mahoney MH, Miller CJ, Nair DT, Politi KA, Worcester KN, Setton RA, Dipiazza R, Sherman EA, Eastman JT, Francklyn C, Robey-Bond S, Rider NL, Gabriel S, Morton DH, Strauss KA (2012). Genetic mapping and exome sequencing identify variants associated with five novel diseases. PLoS One.

[10] Khateb S, Zelinger L, Mizrahi-Meissonnier L, Ayuso C, Koenekoop RK, Laxer U, Gross M, Banin E, Sharon D: **A homozygous nonsense CEP250 mutation combined with a heterozygous nonsense C2orf71 mutation is associated with atypical Usher syndrome.** *J Med Genet* 2014, **ᅟ:**ᅟ. Epub ahead of print. 10.1136/jmedgenet-2014-102287 24780881

[11] Kremer H, van Wijk E, Märker T, Wolfrum U, Roepman R: **Usher syndrome:molecular links of pathogenesis, proteins and pathway.** *Hum Mol Genet* 2006, **15**. ᅟ. Spec No:R262-70. 10.1093/hmg/ddl205 16987892

[12] Michalski N, Michel V, Bahloul A, Lefèvre G, Barral J, Yagi H, Chardenoux S, Weil D, Martin P, Hardelin JP, Sato M, Petit C (2007). Molecular characterization of the ankle-link complex in cochlear hair cells and its role in the hair bundle functioning. J Neurosci.

[13] Grati M, Shin JB, Weston MD, Green J, Bhat MA, Gillespie PG, Kachar B (2012). Localization of PDZD7 to the stereocilia ankle-link associates this scaffolding protein with the Usher syndrome protein network. J Neurosci.

[14] Bahloul A, Simmler MC, Michel V, Leibovici M, Perfettini I, Roux I, Weil D, Nouaille S, Zuo J, Zadro C, Licastro D, Gasparini P, Avan P, Hardelin JP, Petit C (2009). Vezatin, an integral membrane protein of adherens junctions, is required for the sound resilience of cochlear hair cells. EMBO Mol Med.

[15] Kazmierczak P, Sakaguchi H, Tokita J, Wilson-Kubalek EM, Milligan RA, Müller U, Kachar B (2007). Cadherin 23 and protocadherin 15 interact to form tip-link filaments in sensory hair cells. Nature.

[16] El-Amraoui A, Petit C (2010). Cadherins as targets for genetic diseases. Cold Spring Harb Perspect Biol.

[17] Muller U (2008). Cadherins and mechanotransduction by hair cells. Curr Opin Cell Biol.

[18] Sakaguchi H, Tokita J, Muller U, Kachar B (2009). Tip links in hair cells: molecular composition and role in hearing loss. Curr Opin Otolaryngol Head Neck Surg.

[19] Wang A, Liang Y, Fridell RA, Probst FJ, Wilcox ER, Touchman JW, Morton CC, Morell RJ, Noben-Trauth K, Camper SA, Friedman TB (1998). Association of unconventional myosin MYO15 mutations with human nonsyndromic deafness DFNB3. Science.

[20] Belyantseva IA, Boger ET, Naz S, Frolenkov GI, Sellers JR, Ahmed ZM, Griffith AJ, Friedman TB (2005). Myosin-XVa is required for tip localization of whirlin and differential elongation of hair-cell stereocilia. Nat Cell Biol.

[21] Delprat B, Michel V, Goodyear R, Yamasaki Y, Michalski N, El-Amraoui A, Perfettini I, Legrain P, Richardson G, Hardelin JP, Petit C (2005). Myosin XVa and whirlin, two deafness gene products required for hair bundle growth, are located at the stereocilia tips and interact directly. Hum Mol Genet.

[22] Maerker T, van Wijk E, Overlack N, Kersten FF, McGee J, Goldmann T, Sehn E, Roepman R, Walsh EJ, Kremer H, Wolfrum U (2008). A novel Usher protein network at the periciliary reloading point between molecular transport machineries in vertebrate photoreceptor cells. Hum Mol Genet.

[23] Sahly I, Dufour E, Schietroma C, Michel V, Bahloul A, Perfettini I, Pepermans E, Estivalet A, Carette D, Aghaie A, Ebermann I, Lelli A, Iribarne M, Hardelin JP, Weil D, Sahel JA, El-Amraoui A, Petit C (2012). Localization of Usher 1 proteins to the photoreceptor calyceal processes, which are absent from mice. J Cell Biol.

[24] Cosgrove D, Zallocchi M (2014). Usher protein functions in hair cells and photoreceptors. Int J Biochem Cell Biol.

[25] Bonnet C, Grati M, Marlin S, Levilliers J, Hardelin JP, Parodi M, Niasme-Grare M, Zelenika D, Délépine M, Feldmann D, Jonard L, El-Amraoui A, Weil D, Delobel B, Vincent C, Dollfus H, Eliot MM, David A, Calais C, Vigneron J, Montaut-Verient B, Bonneau D, Dubin J, Thauvin C, Duvillard A, Francannet C, Mom T, Lacombe D, Duriez F, Drouin-Garraud V (2011). Complete exon sequencing of all known Usher syndrome genes greatly improves molecular diagnosis. Orphanet J Rare Dis.

[26] Cremers FP, Kimberling WJ, Külm M, de Brouwer AP, van Wijk E, te Brinke H, Cremers CW, Hoefsloot LH, Banfi S, Simonelli F, Fleischhauer JC, Berger W, Kelley PM, Haralambous E, Bitner-Glindzicz M, Webster AR, Saihan Z, De Baere E, Leroy BP, Silvestri G, McKay GJ, Koenekoop RK, Millan JM, Rosenberg T, Joensuu T, Sankila EM, Weil D, Weston MD, Wissinger B, Kremer H (2007). Development of a genotyping microarray for Usher syndrome. J Med Genet.

[27] Jaijo T, Aller E, García-García G, Aparisi MJ, Bernal S, Avila-Fernández A, Barragán I, Baiget M, Ayuso C, Antiñolo G, Díaz-Llopis M, Külm M, Beneyto M, Nájera C, Millán JM (2010). Microarray-based mutation analysis of 183 Spanish families with Usher syndrome. Invest Ophthalmol Vis Sci.

[28] Vozzi D, Aaspõllu A, Athanasakis E, Berto A, Fabretto A, Licastro D, Külm M, Testa F, Trevisi P, Vahter M, Ziviello C, Martini A, Simonelli F, Banfi S, Gasparini P (2011). Molecular epidemiology of Usher syndrome in Italy. Mol Vis.

[29] Aller E, Jaijo T, García-García G, Aparisi MJ, Blesa D, Díaz-Llopis M, Ayuso C, Millán JM (2010). Identification of large rearrangements of the PCDH15 gene by combined MLPA and a CGH: large duplications are responsible for Usher syndrome. Invest Ophthalmol Vis Sci.

[30] Roux AF, Faugère V, Vaché C, Baux D, Besnard T, Léonard S, Blanchet C, Hamel C, Mondain M, Gilbert-Dussardier B, Edery P, Lacombe D, Bonneau D, Holder-Espinasse M, Ambrosetti U, Journel H, David A, Lina-Granade G, Malcolm S, Claustres M (2011). Four-year follow up of diagnostic service in USH1 patients. Invest Ophthalmol Vis Sci.

[31] Steele-Stallard HB, Le Quesne SP, Lenassi E, Luxon LM, Claustres M, Roux AF, Webster AR, Bitner-Glindzicz M (2013). Screening for duplications, deletions and a common intronic mutation detects 35% of second mutations in patients with USH2A monoallelic mutations on Sanger sequencing. Orphanet J Rare Dis.

[32] Choi BY, Park G, Gim J, Kim AR, Kim BJ, Kim HS, Park JH, Park T, Oh SH, Han KH, Park WY (2013). Diagnostic application of targeted resequencing for familial nonsyndromic hearing loss. PLoS One.

[33] Mutai H, Suzuki N, Shimizu A, Torii C, Namba K, Morimoto N, Kudoh J, Kaga K, Kosaki K, Matsunaga T (2013). Diverse spectrum of rare deafness genes underlies early-childhood hearing loss in Japanese patients: a cross-sectional, multi-center next-generation sequencing study. Orphanet J Rare Dis.

[34] Fu Q, Wang F, Wang H, Xu F, Zaneveld JE, Ren H, Keser V, Lopez I, Tuan HF, Salvo JS, Wang X, Zhao L, Wang K, Li Y, Koenekoop RK, Chen R, Sui R (2013). Next-generation sequencing-based molecular diagnosis of a Chinese patient cohort with autosomal recessive retinitis pigmentosa. Invest Ophthalmol Vis Sci.

[35] Glöckle N, Kohl S, Mohr J, Scheurenbrand T, Sprecher A, Weisschuh N, Bernd A, Rudolph G, Schubach M, Poloschek C, Zrenner E, Biskup S, Berger W, Wissinger B, Neidhardt J (2014). Panel-based next generation sequencing as a reliable and efficient technique to detect mutations in unselected patients with retinal dystrophies. Eur J Hum Genet.

[36] Licastro D, Mutarelli M, Peluso I, Neveling K, Wieskamp N, Rispoli R, Vozzi D, Athanasakis E, D’Eustacchio A, Pizzo M, D’Amico F, Ziviello C, Simonelli F, Fabretto A, Scheffer H, Gasparini P, Banfi S, Nigro V (2012). Molecular diagnosis of Usher syndrome: application of two different next generation sequencing-based procedures. PLoS One.

[37] Besnard T, García-García G, Baux D, Vaché C, Faugère V, Larrieu L, Léonard S, Millan JM, Malcolm S, Claustres M, Roux AF (2014). Experience of targeted Usher exome sequencing as a clinical test. Mol Genet Genomic Med.

[38] Yoshimura H, Iwasaki S, Nishio SY, Kumakawa K, Tono T, Kobayashi Y, Sato H, Nagai K, Ishikawa K, Ikezono T, Naito Y, Fukushima K, Oshikawa C, Kimitsuki T, Nakanishi H, Usami S (2014). Massively parallel DNA sequencing facilitates diagnosis of patients with Usher syndrome type 1. PLoS One.

[39] Rong W, Chen X, Zhao K, Liu Y, Liu X, Ha S, Liu W, Kang X, Sheng X, Zhao C (2014). Novel and recurrent MYO7A mutations in Usher syndrome type 1 and type 2. PLoS One.

[40] von Brederlow B, Bolz H, Janecke A, La O, Cabrera A, Rudolph G, Lorenz B, Schwinger E, Gal A (2002). Identification and in vitro expression of novel CDH23 mutations of patients with Usher syndrome type 1D. Hum Mutat.

[41] Garcia-Garcia G, Aparisi MJ, Jaijo T, Rodrigo R, Leon AM, Avila-Fernandez A, Blanco-Kelly F, Bernal S, Navarro R, Diaz-Llopis M, Baiget M, Ayuso C, Millan JM, Aller E (2011). Mutational screening of the USH2A gene in Spanish USH patients reveals 23 novel pathogenic mutations. Orphanet J Rare Dis.

[42] Schultz JM, Bhatti R, Madeo AC, Turriff A, Muskett JA, Zalewski CK, King KA, Ahmed ZM, Riazuddin S, Ahmad N, Hussain Z, Qasim M, Kahn SN, Meltzer MR, Liu XZ, Munisamy M, Ghosh M, Rehm HL, Tsilou ET, Griffith AJ, Zein WM, Brewer CC, Riazuddin S, Friedman TB (2011). Allelic hierarchy of CDH23 mutations causing non-syndromic deafness DFNB12 or Usher syndrome USH1D in compound heterozygotes. J Med Genet.

[43] Roux AF, Faugère V, Le Guédard S, Pallares-Ruiz N, Vielle A, Chambert S, Marlin S, Hamel C, Gilbert B, Malcolm S, Claustres M, French Usher Syndrome Collaboration (2006). Survey of the frequency of USH1 gene mutations in a cohort of Usher patients shows the importance of cadherin 23 and protocadherin 15 genes and establishes a detection rate of above 90%. J Med Genet.

[44] Ahmed ZM, Riazuddin S, Bernstein SL, Ahmed Z, Khan S, Griffith AJ, Morell RJ, Friedman TB, Riazuddin S, Wilcox ER (2001). Mutations of the protocadherin gene PCDH15 cause Usher syndrome type 1 F. Am J Hum Genet.

[45] Jaijo T, Oshima A, Aller E, Carney C, Usami S, Millán JM, Kimberling WJ (2012). Mutation screening of the PCDH15 gene in Spanish patients with Usher syndrome type I. Mol Vis.

[46] Riazuddin S, Nazli S, Ahmed ZM, Yang Y, Zulfiqar F, Shaikh RS, Zafar AU, Khan SN, Sabar F, Javid FT, Wilcox ER, Tsilou E, Boger ET, Sellers JR, Belyantseva IA, Riazuddin S, Friedman TB (2008). Mutation spectrum of MYO7A and evaluation of a novel nonsyndromic deafness DFNB2 allele with residual function. Hum Mutat.

[47] Liu XZ, Hope C, Liang CY, Zou JM, Xu LR, Cole T, Mueller RF, Bundey S, Nance W, Steel KP, Brown SD (1999). A mutation (2314delG) in the Usher syndrome type IIA gene: high prevalence and phenotypic variation. Am J Hum Genet.

[48] Bharadwaj AK, Kasztejna JP, Huq S, Berson EL, Dryja TP (2000). Evaluation of the myosin VIIA gene and visual function in patients with Usher syndrome type I. Exp Eye Res.

[49] Aller E, Jaijo T, Beneyto M, Nájera C, Oltra S, Ayuso C, Baiget M, Carballo M, Antiñolo G, Valverde D, Moreno F, Vilela C, Collado D, Pérez-Garrigues H, Navea A, Millán JM (2006). Identification of 14 novel mutations in the long isoform of USH2A in Spanish patients with Usher syndrome type II. J Med Genet.

[50] Le Quesne SP, Saihan Z, Rangesh N, Steele-Stallard HB, Ambrose J, Coffey A, Emmerson J, Haralambous E, Hughes Y, Steel KP, Luxon LM, Webster AR, Bitner-Glindzicz M (2012). Comprehensive sequence analysis of nine Usher syndrome genes in the UK National Collaborative Usher Study. J Med Genet.

[51] Ouyang XM, Yan D, Du LL, Hejtmancik JF, Jacobson SG, Nance WE, Li AR, Angeli S, Kaiser M, Newton V, Brown SD, Balkany T, Liu XZ (2005). Characterization of Usher syndrome type I gene mutations in an Usher syndrome patient population. Hum Genet.

[52] Besnard T, Vaché C, Baux D, Larrieu L, Abadie C, Blanchet C, Odent S, Blanchet P, Calvas P, Hamel C, Dollfus H, Lina-Granade G, Lespinasse J, David A, Isidor B, Morin G, Malcolm S, Tuffery-Giraud S, Claustres M, Roux AF (2012). Non-USH2A mutations in USH2 patients. Hum Mutat.

[53] Oshima A, Jaijo T, Aller E, Millan JM, Carney C, Usami S, Moller C, Kimberling WJ (2008). Mutation profile of the CDH23 gene in 56 probands with Usher syndrome type I. Hum Mutat.

[54] Janecke AR, Meins M, Sadeghi M, Grundmann K, Apfelstedt-Sylla E, Zrenner E, Rosenberg T, Gal A (1999). Twelve novel myosin VIIA mutations in 34 patients with Usher syndrome type I: confirmation of genetic heterogeneity. Hum Mutat.

[55] Bernal S, Medà C, Solans T, Ayuso C, Garcia-Sandoval B, Valverde D, Del Rio E, Baiget M (2005). Clinical and genetic studies in Spanish patients with Usher syndrome type II: description of new mutations and evidence for a lack of genotype—phenotype correlation. Clin Genet.

[56] Jacobson SG, Aleman TS, Sumaroka A, Cideciyan AV, Roman AJ, Windsor EA, Schwartz SB, Rehm HL, Kimberling WJ (2009). Disease boundaries in the retina of patients with Usher syndrome caused by MYO7A gene mutations. Invest Ophthalmol Vis Sci.

[57] Kaiserman N, Obolensky A, Banin E, Sharon D (2007). Novel USH2A mutations in Israeli patients with retinitis pigmentosa and Usher syndrome type 2. Arch Ophthalmol.

[58] Vaché C, Besnard T, le Berre P, García-García G, Baux D, Larrieu L, Abadie C, Blanchet C, Bolz HJ, Millan J, Hamel C, Malcolm S, Claustres M, Roux AF (2012). Usher syndrome type 2 caused by activation of an USH2A pseudoexon: implications for diagnosis and therapy. Hum Mutat.

[59] Ouyang XM, Xia XJ, Verpy E, Du LL, Pandya A, Petit C, Balkany T, Nance WE, Liu XZ (2002). Mutations in the alternatively spliced exons of USH1C cause non syndromic recessive deafness. Hum Genet.

[60] Jaijo T, Aller E, Beneyto M, Najera C, Graziano C, Turchetti D, Seri M, Ayuso C, Baiget M, Moreno F, Morera C, Perez-Garrigues H, Millan JM (2007). MYO7A mutation screening in Usher syndrome type I patients from diverse origins. J Med Genet.

[61] Ross M, Russ C, Costello M, Hollinger A, Lennon NJ, Hegarty R, Nusbaum C, Jaffe DB (2013). Characterizing and measuring bias in sequence data. Genome Biol.

[62] Le Guédard S, Faugère V, Malcolm S, Claustres M, Roux AF (2007). Large genomicrearrangements within the PCDH15 gene are a significant cause of USH1F syndrome. Mol Vis.

[63] Eisenberger T, Neuhaus C, Khan AO, Decker C, Preising MN, Friedburg C, Bieg A, Gliem M, Charbel Issa P, Holz FG, Baig SM, Hellenbroich Y, Galvez A, Platzer K, Wollnik B, Laddach N, Ghaffari SR, Rafati M, Botzenhart E, Tinschert S, Börger D, Bohring A, Schreml J, Körtge-Jung S, Schell-Apacik C, Bakur K, Al-Aama JY, Neuhann T, Herkenrath P, Nürnberg G (2013). Increasing the yield in targeted next-generation sequencing by implicating CNV analysis, non-coding exons and the overall variant load: the example of retinal dystrophies. PLoS One.

